# Epidemics on hypergraphs: spectral thresholds for extinction

**DOI:** 10.1098/rspa.2021.0232

**Published:** 2021-08

**Authors:** Desmond J. Higham, Henry-Louis de Kergorlay

**Affiliations:** School of Mathematics, University of Edinburgh, Edinburgh EH9 3FD, UK

**Keywords:** susceptible–infected–susceptible, Markov process, extinction, stability

## Abstract

Epidemic spreading is well understood when a disease propagates around a contact graph. In a stochastic susceptible–infected–susceptible setting, spectral conditions characterize whether the disease vanishes. However, modelling human interactions using a graph is a simplification which only considers pairwise relationships. This does not fully represent the more realistic case where people meet in groups. Hyperedges can be used to record higher order interactions, yielding more faithful and flexible models and allowing for the rate of infection of a node to depend on group size and also to vary as a nonlinear function of the number of infectious neighbours. We discuss different types of contagion models in this hypergraph setting and derive spectral conditions that characterize whether the disease vanishes. We study both the exact individual-level stochastic model and a deterministic mean field ODE approximation. Numerical simulations are provided to illustrate the analysis. We also interpret our results and show how the hypergraph model allows us to distinguish between contributions to infectiousness that (i) are inherent in the nature of the pathogen and (ii) arise from behavioural choices (such as social distancing, increased hygiene and use of masks). This raises the possibility of more accurately quantifying the effect of interventions that are designed to contain the spread of a virus.

## 1. Introduction

Compartmental models for disease propagation have a long and illustrious history [1,2], and they remain a fundamental predictive tool [3,4]. For a stochastic, individual-level model, it has been suggested recently that hyperedge information should be incorporated [5–7]. Hyperedges allow us to account directly for group interactions of any size, rather than, as in, for example, [8–10], treating them as a collection of essentially independent pairwise encounters.

In this work, we contribute to the modelling and analysis of disease spreading on a hypergraph. We include the case where the number of infected nodes in a hyperedge contributes nonlinearly to the overall infection rate; this covers the so-called *collective contagion model* setting and a new alternative that we call a *collective suppression model*. The main contributions of our work are

— a mean field approximation ((4.1) and (4.2)) with a spectral condition for local asymptotic stability of the zero-infection state (theorem 6.1) and an extension to global asymptotic stability (theorem 6.5) when the nonlinear infection function is concave,
— for the exact, individual-level model, a spectral condition for exponential decay of the non-extinction probability in the concave case (theorem 8.1) and a spectral bound on the expected time to extinction (corollary 8.2),
— extensions of these results to more general partitioned hypergraph models, where distinct infection rates apply to different categories of hyperedge (theorems 6.2, 6.6 and 8.3 and corollary 8.4),
— results for the non-concave collective contagion model (theorems 9.1 and 9.2),
— a complementary condition that rules out extinction of the disease (theorem 8.6),
— interpretations of these mathematical results: the spectral thresholds for disease extinction naturally distinguish between the inherent biological infectiousness of the disease and behavioural choices of the individuals in the population, allowing us to account for intervention strategies (§10).

The paper is organized as follows. In §2, we introduce the traditional graph-based susceptible–infected–susceptible (SIS) model and quote a spectral condition that characterizes control of the disease. We then discuss the generalization to hyperedges, and motivate the use of infection rates that do not scale linearly with respect to the number of infected neighbours. Section 3 formalizes the hypergraph model and shows how it may be simulated. In §4, we derive a mean field approximation to characterize the behaviour of the model, and in §5 we give computational results to illustrate its relevance. Section 6 analyses the deterministic mean field setting and gives a spectral condition for long-term decay of the disease. The result is local for general infection rates and global (independent of the initial condition) for the concave case. This spectral condition generalizes a well-known result concerning disease propagation on a graph. Section 7 provides further computational simulations to illustrate the spectral threshold. The full stochastic model is then studied in §8, where we extend the analysis in [8] to our hypergraph setting. Here, we study extinction of the disease in the case where the nonlinearity in the infection rate is concave. We also derive conditions for non-extinction. In §9, we extend our analysis to the so-called collective contagion model proposed in [5–7]. In §10, we summarize and interpret our results and discuss follow-on work.

For a review of recent studies of spreading processes on hypergraphs, including the dissemination of rumours, opinions and knowledge, we recommend [11, §7.1.2]. The model that we study fits into the framework of [12]. This work introduced the idea of a nonlinear ‘infection pressure’ from each hyperedge, and derived a mean field approximation that was compared with microscale-level simulation results. In [6], the authors studied this type of model on simplicial complexes of degree up to 2 (a subclass of the more general hypergraph setting) and also studied a mean field approximation. These authors examined the mean field system from a dynamical systems perspective and analysed issues such as bistability, hysteresis and discontinuous transitions. Similarly, in [5,7], a hypergraph version was considered. Our work differs from these studies in (i) focusing on the derivation of spectral thresholds for extinction of a disease in both the exact and mean field settings and (ii) seeking to interpret the results from a mathematical modelling perspective. We mention that it would also be of interest to develop corresponding thresholds for the mean field models in [5,6,12].

## 2. Stochastic susceptible–infected–susceptible models

### (a) Stochastic susceptible–infected–susceptible model on a graph

Classical ODE compartmental models are based on the assumption that any pair of individuals is equally likely to interact—this is the homogeneous mixing case [1]. If, instead, we have knowledge of all possible pairwise interactions between individuals, then this information may be incorporated via a contact graph and used in a stochastic model. Here, each node represents an individual, and an edge between nodes *i* and *j* indicates that individuals *i* and *j* interact. For a population with *n* individuals, we may let $A\in R^{n\times n}$ denote the corresponding symmetric adjacency matrix, so nodes *i* and *j* interact if and only if $A_{ij}=1$. In this setting, a stochastic SIS model uses the two-state random variable $X_{i}(t)$ to represent the status of node *i* at time *t*, with $X_{i}(t)=0$ for a susceptible node and $X_{i}(t)=1$ for an infected node. Each $X_{i}(t)$ then follows a continuous time Markov process where the infection rate is given by

2.1 $$\beta \sum_{j=1}^{n}A_{ij}X_{j}(t)$$

and the recovery rate is *δ*. Here, β>0 and δ>0 are parameters governing the strength of the two effects. In this model, we see from (2.1) that the current chance of infection increases linearly in proportion to the current number of infected neighbours.

This model was studied in [10, theorem 1], where it was argued that the condition

2.2 $$\lambda (A)\frac{\beta }{\delta }<1$$

guarantees that the disease will die out. Here, λ(A) denotes the largest eigenvalue of the symmetric matrix A. Further justification for this result may be found, for example, in [8,9]. We note that (2.2) gives an elegant generalization of the homogeneous mixing case (where *A* corresponds to the complete graph).

### (b) Why use a hypergraph?

It has been argued [11,13–16] that in many network science applications we lose information by recording only pairwise interactions. For example, e-mails can be sent to groups of recipients, scholarly articles may have multiple coauthors and many proteins may interact to form a complex. In such cases, recording the relevant lists of interacting nodes gives a more informative picture than reducing these down to a collection of edges.

In the setting of an SIS model, we may argue that individuals typically come together in well-defined groups; for example, in a household, a workplace or a social setting. Such groups may be handled by the use of hyperedges, leading to a hypergraph; these concepts are formalized in the next section.

With a classic graph model, as described in §2a, all types of (pairwise) interactions are treated equally and the rate of infection of a node is linearly proportional to the number of infectious neighbours. With a hypergraph we may consider more intricate contagion mechanisms. For example, using the terminology of [7], the *collective contagion* model is studied in [5–7]. Here, infection only starts spreading within a hyperedge after a certain critical number of infectious neighbours has been reached. This type of behaviour is relevant, for example, in a shared office area where infection occurs only when a minimal viral load is exceeded. The critical number may also depend on the environment and group size; in a given office space a small number of workers may be able to socially distance in a way that effectively eliminates the risk of infection. Hence, we may wish to use a different threshold for different sizes and categories of hyperedge.

We mention here that an alternative type of mechanism may also operate, which we call *collective suppression*. Imagine that a disease may be contracted through contact with a surface that was previously touched by an infected individual. Now suppose that a group of individuals is likely to use the same physical object, such as a door handle, hand rail, cash machine or water cooler. If an infected individual contaminates the object, then further contamination by other individuals is less relevant. In this case, doubling the number of infected users will increase the risk by a factor less than 2; generally, risk grows sublinearly as a function of the size of the hyperedge.

These arguments motivate us to allow the rate of infection of a node within a hyperdege to depend on a generic function *f* of the number of infectious neighbours in that hyperedge; this approach was also taken in [12]. The arguments also motivate us to study the case where the form of the nonlinearity depends on the type of hyperedge. We will be particularly concerned with the case where *f* is concave, since this is tractable for analysis and allows us to draw conclusions about the collective contagion model. We note that if *f* is the identity, then we recover linear dependence on the number of infectious neighbours and the hypergraph model is equivalent to a virus spreading on the clique graph of the hypergraph.

## 3. Susceptible–infected–susceptible on a hypergraph

### (a) Background

We continue with some standard definitions [17].

**Definition 3.1.**

A *hypergraph* is a tuple H:=(V,E) of *nodes* *V* and *hyperedges* *E* such that E⊂P(V). Here, P(V) denotes the power set of *V*.

We will let *n* and *m* denote the number of nodes and hyperedges, respectively; that is, |V|=n and |E|=m. Loosely, a hypergraph generalizes the concept of a graph by allowing an ‘edge’ to be a list of more than two nodes.

**Definition 3.2.**

Consider a hypergraph H:=(V,E). The *incidence matrix*, I, is the n×m matrix such that $I_{ih}=1$ if node *i* belongs to hyperedge h and $I_{ih}=0$ otherwise.

It is also useful to introduce $W:=II^{T}$. This n×n matrix has the property that $W_{ij}$ records the number of hyperedges containing both nodes *i* and *j*. In particular, if H is a graph then *W* is the affinity matrix of the graph.

### (b) General infection model

In our context, the nodes represent individuals and a hyperedge records a collection of individuals who are known to interact as a group. As in the graph case introduced in §2a, we use a state vector X(t), which follows a continuous time Markov process, where, for each 1≤i≤n, $X_{i}(t)=1$ if node *i* is infected at time *t* and $X_{i}(t)=0$ otherwise. We continue to assume that an infectious node becomes susceptible with constant recovery rate δ>0. However, generalizing (2.1), we now assume that a susceptible node *i* becomes infectious with rate

3.1 $$\beta \sum_{h\in E}I_{ih}f\left(\sum_{j=1}^{n}X_{j}(t)I_{jh}\right),$$

where β>0 is a constant. In (3.1), $f:R_{+}\to R_{+}$ specifies the manner in which the contribution to the overall level of infectiousness from each hyperedge involving node *i* is assumed to increase in proportion to the number of infected nodes in that hyperedge. Throughout our analysis, we will always assume that f(0)=0 and *f* is $C^{1}$ in a neighbourhood of 0.

If *f* is the identity, the rate of infection reduces to $\beta \sum_{j=1}^{n}W_{ij}X_{j}(t)$. This gives a weighted version of the infection rate of an SIS model on a graph. As discussed in §2b, it may be appropriate to choose nonlinear *f* in certain circumstances. We note that in [12] the authors have in mind functions which behave like the identity near the origin and have a horizontal asymptote. Instances of such functions are x↦arctan(x) and x↦min{x,c} for some c>0. Relaxing these conditions, we may ask more generally in such a setting that the function be concave. On the other hand, the authors in [5,6] consider a *collective contagion model*, where infection spreads within a hyperdge only if a certain threshold of infectious vertices is reached in that hyperedge. A collective contagion model may be represented via the function $x\mapsto c_{2}1(x\geq c_{1})$ for some $c_{1},c_{2}>0$, or x↦max{0,x−c} for some c>0.

### (c) Partitioned hypergraph model

Following the discussion in §2b, we also introduce a more general case where we partition the hyperedges into *K* disjoint categories with each category 1≤k≤K having its own distinct rate of infection in response to the number of infected nodes in a hyperedge, represented by a function $f_{k}$. For example, the categories may correspond to different types of housing, workplaces, hospitality venues or sports facilities, each representing different physical spaces and forms of interaction. We may then represent the infection rate of node *i* as

3.2 $$\beta \sum_{k=1}^{K}\sum_{h\in E}I_{ih}^{\left(k\right)}f_{k}\left(\sum_{j=1}^{n}X_{j}(t)I_{jh}^{\left(k\right)}\right),$$

where we let $I_{ih}^{\left(k\right)}=1$ if *i* belongs to hyperedge *h* in the category *k* and $I_{ih}^{\left(k\right)}=0$ otherwise; so $I^{\left(k\right)}$ is the incidence matrix of the subhypergraph consisting of only the hyperedges from category *k*. We will refer to this as a *partitioned hypergraph model*.

In this generalized case, a collective contagion model could be defined by first organizing the hyperedges into categories depending on their size, so that category *k* is the set of hyperedges of size k+1. A collective contagion model may then be represented, for example, via the functions $f_{1}:x\mapsto x$ and $f_{k}:x\mapsto c_{2,k}1(x\geq c_{1,k})$, k∈{2,…,K}.

## 4. Mean field approximation

A classic approach to studying processes with random infection rates is to develop a mean field approximation for the expected process

$${\left(E[X_{i}(t)]\right)}_{t\geq 0}={\left(P(X_{i}(t)=1)\right)}_{t\geq 0}=:{\left(p_{i}(t)\right)}_{t\geq 0},$$

with deterministic rates. For the model in §3b, $X_{i}(t)\in \{0,1\}$ and we have $X_{i}(t)\to X_{i}(t)+1$ with rate given by (3.1) if and only if $X_{i}(t)=0$. Conversely we have $X_{i}(t)\to X_{i}(t)-1$ with rate *δ* if and only if $X_{i}(t)=1$. Making the simplifying assumption that the infection rate for node *i* is independent of its state, we have

$$\frac{\text{d}p_{i}(t)}{\text{d}t}=E\left[\beta \sum_{h\in E}I_{ih}f\left(\sum_{j=1}^{n}X_{j}(t)I_{jh}\right)\right]P(X_{i}(t)=0)-\delta \;P(X_{i}(t)=1).$$

Now, in order to obtain a model that just involves $p_{i}(t)$, we introduce a further approximation by interchanging the application of *f* and the expectation in the first factor on the right-hand side. We arrive at the deterministic mean field ODE

4.1 $$\frac{\text{d}P(t)}{\text{d}t}=g(P(t)),$$

where $g_{i}:R^{n}\to R$ is defined by

4.2 $$g_{i}(P(t)):=\beta \sum_{h\in E}I_{ih}f\left(\sum_{j=1}^{n}p_{j}(t)I_{jh}\right)(1-p_{i}(t))-\delta p_{i}(t).$$

## 5. Simulations and comparison between exact and mean field models

Let us emphasize that the approximate infection rates in (4.2) differ in general from the expectation of the random rates in (3.1). When the function *f* is concave, however, Jensen’s reverse inequality indicates that the rates in (4.2) are greater than the expectation of the rates in (3.1). Hence, in this case the expected quantities $p_{i}(t)$ are overestimated by (4.1) and (4.2). This is fine when we are looking for conditions for the disease to vanish. If *f* is not concave (e.g. for a collective contagion model) it is less clear *a priori* how the exact model and mean field ODE compare. In this section, we therefore present results of computational simulations in order to gain insight into the accuracy of our mean field approximation.

### (a) Simulation algorithm

Before presenting numerical results, we summarize our approach for simulating the individual-level stochastic model, which is based on a standard time discretization; see, for example, [12]. Using a small fixed time step Δt, we advance from time *t* to t+Δt as follows. First, let $r\in {\left[0,1\right]}^{n}$ be a random vector of i.i.d. values uniformly sampled from [0,1]. For every node 1≤i≤n,

— when $X_{i}(t)=0$, set $X_{i}(t+\Delta t)=1$ if
  $$r_{i}<1-\exp\left(-\beta \sum_{h}I_{ih}f\left(\sum_{j}X_{j}(t)I_{jh}\right)\Delta t\right),$$
  and set $X_{i}(t+\Delta t)=0$ otherwise;
— when $X_{i}(t)=1$, set $X_{i}(t+\Delta t)=0$ if
  $$r_{i}<1-\exp(-\delta \Delta t),$$
  and set $X_{i}(t+\Delta t)=1$ otherwise.

### (b) Computational results

In the simulations, we chose n=400 nodes with fixed recovery rate δ=1 and a time step of Δt=0.05. We look at results for different choices of (i) the infection strength, *β*, and (ii) the (independent) initial probability for each node to be infectious, which we denote $i_{0}$. We simulated the mean field ODE using Euler’s method with time step of 0.05. The largest size of a hyperedge was 5 and we distributed the number of hyperedges for the hypergraph randomly as follows: 300 edges, 200 hyperedges of size 3, 100 hyperedges of size 4 and 50 hyperedges of size 5. To give a feel for the level of fluctuation, the individual-level paths are averaged over 10 runs, each with the same hypergraph connectivity and initial state.

**Figure 1.**
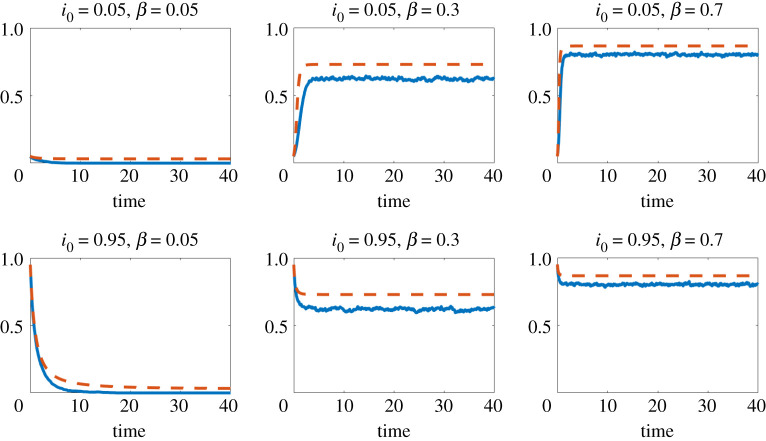
Here, f(x)=min(x,3). Red dashed line: mean field approximation from (4.1) to (4.2). Blue solid line: proportion of infected individuals, $\sum_{i}X_{i}(t)/n$, from the individual-level stochastic model (3.1), averaged over 10 runs.(Online version in colour.)

**Figure 2.**
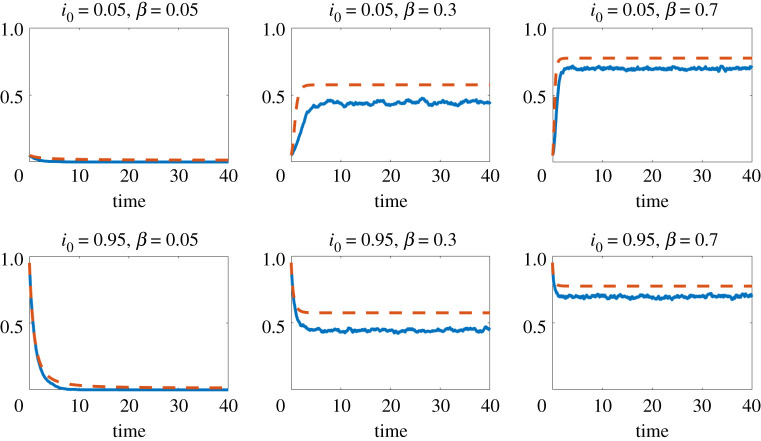
Here, f(x)=log⁡(1+x). Red dashed line: mean field approximation from (4.1) to (4.2). Blue solid line: proportion of infected individuals, $\sum_{i}X_{i}(t)/n$, from the individual-level stochastic model (3.1), averaged over 10 runs.(Online version in colour.)

**Figure 3.**
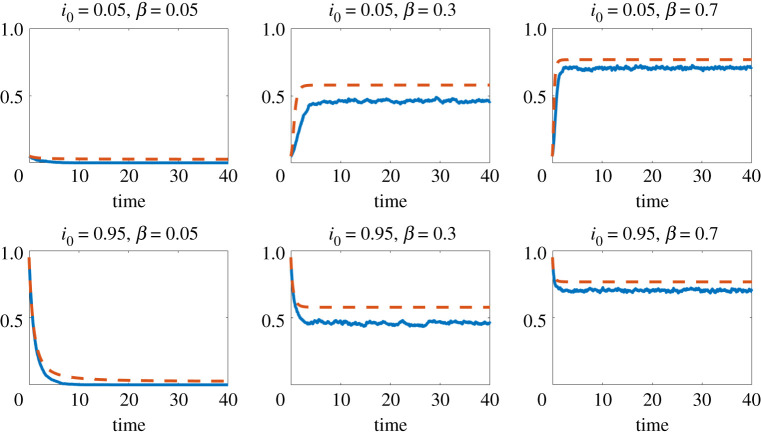
Here, f(x)=arctan(x). Red dashed line: mean field approximation from (4.1) to (4.2). Blue solid line: proportion of infected individuals, $\sum_{i}X_{i}(t)/n$, from the individual-level stochastic model (3.1), averaged over 10 runs.(Online version in colour.)

Figures 1–3 show results for three concave choices of *f*; respectively,

— f(x)=min(x,3),
— f(x)=log⁡(1+x),
— f(x)=arctan(x).

**Figure 4.**
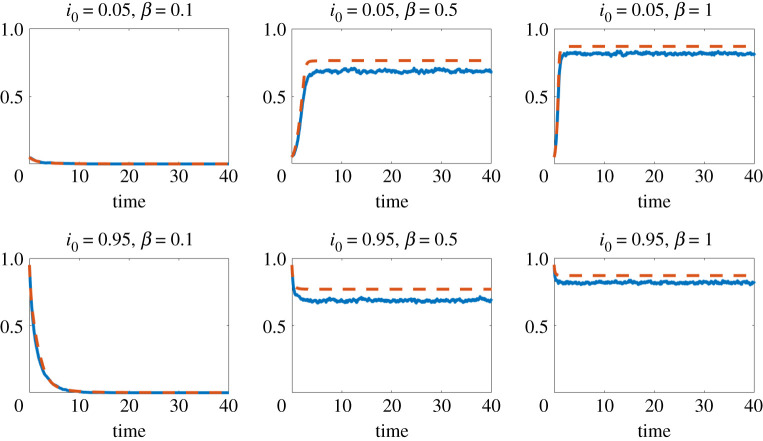
Collective contagion model on a partitioned hypergraph. Red dashed line: mean field approximation from (4.1) with (6.2). Blue solid line: proportion of infected individuals, $\sum_{i}X_{i}(t)/n$, from the individual-level stochastic model (3.1), averaged over 10 runs.(Online version in colour.)

For figure 4, we used a collective contagion model on a partitioned hypergraph. Assigning each hyperedge to a category in {1,2,3,4}, where category *k* contains the hyperedges of size k+1, we chose the following associated functions to determine the infection rates: $f_{1}(x):=x$, and for k∈{2,3,4}, $f_{k}(x):=(k-1)1(x\geq k-1)$.

The four figures show the proportion of infectious individuals as a function of time. In these simulations, and others not reported here, we observe that the initial value $i_{0}$ does not affect the asymptotic behaviour of the process: the process vanishes or converges to a non-zero equilibrium depending on the value of *β* but regardless of the value of $i_{0}$. In figures 1–3, where *f* is concave, we know that the mean field model gives an upper bound on the expected proportion of infected individuals in the microscale model. We also see that the mean field model provides a reasonably sharp approximation. Moreover, we see a similar level of sharpness in figure 4 for the collective contagion model, where *f* is not concave.

A key advantage of the mean field approximation is that it gives rise to a deterministic autonomous dynamical system for which there exists a rich theory to study the asymptotic stability of equilibrium points. This motivates the analysis in the next section.

## 6. Stability analysis

We provide below spectral conditions which imply that the infection-free solution $0\in R^{n}$ is a locally or globally asymptotically stable equilibrium of (4.1) and (4.2). We will find that local asymptotic stability can be shown with no structural assumptions on *f*. We will also find that global asymptotic stability follows *under the same conditions* when *f* is concave. Our conclusions fit into a framework that generalizes the graph case (2.2): the spectral threshold takes the form

$$\lambda (W)\frac{c_{f}\beta }{\delta }<1,$$

for some constant $c_{f}>0$ depending only on the choice of *f*.

Throughout this work, to be concrete we let ||⋅|| denote the Euclidean norm.

### (a) Local asymptotic stability

**Theorem 6.1.**

*If*

6.1 $$\lambda (W)\frac{f^{\prime }(0)\beta }{\delta }<1,$$

*then* $0\in R^{n}$ *is a locally asymptotically stable equilibrium of* (*4.1*) *and* (*4.2*); *that is, there exists a positive γ such that* $||P(0)||<\gamma \Rightarrow \lim_{t\to \infty }||P(t)||=0$.

**Proof.**

We see that g(0)=0, so $0\in R^{n}$ is an equilibrium for (4.1). It remains to show that this solution is locally asymptotically stable. Appealing to a standard linearization result [18], it suffices to show that every eigenvalue of the Jacobian matrix ∇g(0) has a negative real part. We compute

$$\frac{\partial g_{i}}{\partial p_{j_{0}}}=\left\{ \begin{array}{ll} \beta \sum_{h}I_{ih}I_{j_{0}h}f^{\prime }\left(\sum_{j}p_{j}I_{jh}\right)(1-p_{i}), & j_{0}\neq i,\\ \beta \sum_{h}I_{ih}I_{j_{0}h}f^{\prime }\left(\sum_{j}p_{j}I_{jh}\right)(1-p_{i})-\beta \sum_{h}I_{ih}f\left(\sum_{j}p_{j}I_{jh}\right)-\delta , & j_{0}=i. \end{array}\right.$$

We see that $\nabla g(0)=\beta f^{\prime }(0)W-\delta I$. This matrix is symmetric and therefore has real eigenvalues. Hence, it suffices that the largest eigenvalue of $\beta f^{\prime }(0)W$ does not exceed *δ*, and the result follows. ▪

Theorem 6.1 extends to the partitioned model in (3.2). In this case, $g_{i}(P(t))$ in the mean field ODE (4.1) is defined as

6.2 $$g_{i}(P(t)):=\beta \sum_{k=1}^{K}\sum_{h\in E}I_{ih}^{\left(k\right)}f_{k}\left(\sum_{j=1}^{n}p_{j}(t)I_{jh}^{\left(k\right)}\right)(1-p_{i}(t))-\delta p_{i}(t),$$

and we let $W^{\left(k\right)}:=I^{\left(k\right)}{I^{\left(k\right)}}^{T}$.

**Theorem 6.2.**

*If*

6.3 $$\lambda \left(\sum_{k=1}^{K}f_{k}^{\prime }(0)W^{\left(k\right)}\right)\frac{\beta }{\delta }<1,$$

*then* $0\in R^{n}$ *is a locally asymptotically stable equilibrium of* (*4.1*), *with* $g_{i}$ *defined in* (*6.2*).

**Proof.**

The proof of theorem 6.1 extends straightforwardly. We compute

$$\begin{array}{rl} \frac{\partial g_{i}}{\partial p_{j_{0}}} & =\left\{ \begin{array}{ll} \beta \sum_{k}\sum_{h}I_{ih}^{\left(k\right)}I_{j_{0}h}^{\left(k\right)}f_{k}^{\prime }\left(\sum_{j}p_{j}I_{jh}^{\left(k\right)}\right)(1-p_{i}), & j_{0}\neq i,\\ \beta \sum_{k}\sum_{h}I_{ih}^{\left(k\right)}I_{j_{0}h}^{\left(k\right)}f_{k}^{\prime }\left(\sum_{j}p_{j}I_{jh}^{\left(k\right)}\right)(1-p_{i}) & \\ -\beta \sum_{k}\sum_{h}I_{ih}^{\left(k\right)}f_{k}\left(\sum_{j}p_{j}I_{jh}^{\left(k\right)}\right)-\delta , & j_{0}=i, \end{array}\right.\\ & \Rightarrow \frac{\partial g_{i}}{\partial p_{j_{0}}}{|}_{P=0}=\left\{ \begin{array}{ll} \beta \sum_{k}W_{ij}^{\left(k\right)}f_{k}^{\prime }(0), & j_{0}\neq i,\\ \beta \sum_{k}W_{ij}^{\left(k\right)}f_{k}^{\prime }(0)-\delta , & j_{0}=i, \end{array}\right. \end{array}$$

and note that

$$\lambda \left(\beta \sum_{k}f_{k}^{\prime }(0)W^{\left(k\right)}-\delta I\right)<0\Leftrightarrow \lambda \left(\sum_{k}f_{k}^{\prime }(0)W^{\left(k\right)}\right)<\frac{\delta }{\beta }.$$

 ▪

### (b) Global asymptotic stability for the concave infection model

We now show that when *f* is concave the condition in theorem 6.1 ensures global stability of the zero equilibrium, and hence guarantees that the disease dies out according to the mean field approximation.

**Definition 6.3.**

Given a matrix *A*, define its symmetric version to be

$$A^{\left(S\right)}:=\frac{\left(A+A^{T}\right)}{2}.$$

**Lemma 6.4.**

*Suppose that A and B are* n×n *real matrices, and suppose that there exists a diagonal matrix* Λ such that for all $i\in \{1,2,\ldots ,n\},\;\Lambda_{ii}\geq 0$, *and*

A=B−Λ.

*Then the largest eigenvalues of A and B satisfy* λ(A)≤λ(B), *and the largest eigenvalues of* $A^{\left(S\right)}$ *and* $B^{\left(S\right)}$ *also satisfy* $\lambda (A^{\left(S\right)})\leq \lambda (B^{\left(S\right)})$.

**Proof.**

Let x be a unit eigenvector associated with $\lambda (A^{\left(S\right)})$. We have

$$\begin{array}{rl} 2\lambda (A^{\left(S\right)}) & =x^{T}Ax+x^{T}A^{T}x\\ & =x^{T}Bx+x^{T}B^{T}x-2x^{T}\Lambda x\\ & =\sum_{i,j=1}^{n}(b_{ij}+b_{ji})x_{i}x_{j}-2\sum_{i=1}^{n}\Lambda_{ii}x_{i}^{2}\\ & \leq x^{T}(B+B^{T})x\\ & \leq \max\{y^{T}(B+B^{T})y\;|\;y^{T}y=1\}=2\lambda (B^{\left(S\right)}). \end{array}$$

The inequality λ(A)≤λ(B) may be shown similarly. ▪

**Theorem 6.5.**

*Suppose f is concave. If*

$$\lambda (W)\frac{f^{\prime }(0)\beta }{\delta }<1,$$

*then* $0\in R^{n}$ *is a globally asymptotically stable equilibrium of* (*4.1*) *and* (*4.2*); *so* $\lim_{t\to \infty }||P(t)||=0$ *for any valid initial condition* $(\text{that is, with}\;0\leq p{\left(0\right)}_{i}\leq 1).$

**Proof.**

From the global asymptotic stability result in [19, lemma 1′], it is sufficient to show that all eigenvalues of the symmetric matrix ${\left(\nabla g(P)\right)}^{\left(S\right)}$ are strictly less than 0, for all P≠0. We have

$$\begin{array}{rl} \frac{\partial g_{i}}{\partial p_{j_{0}}} & =\left\{ \begin{array}{ll} \beta \sum_{h}I_{ih}I_{j_{0}h}f^{\prime }(\sum_{j}p_{j}I_{jh})(1-p_{i}), & \;j_{0}\neq i,\\ \beta \sum_{h}I_{ih}I_{j_{0}h}f^{\prime }(\sum_{j}p_{j}I_{jh})(1-p_{i})-\beta \sum_{h}I_{ih}f(\sum_{j}p_{j}I_{jh})-\delta , & \;j_{0}=i. \end{array}\right. \end{array}$$

Letting *B* denote the n×n matrix given by

$$B_{ij_{0}}=\left\{ \begin{array}{ll} \beta \sum_{h}I_{ih}I_{j_{0}h}f^{\prime }\left(\sum_{j}p_{j}I_{jh}\right)(1-p_{i}), & j_{0}\neq i,\\ \beta \sum_{h}I_{ih}I_{j_{0}h}f^{\prime }\left(\sum_{j}p_{j}I_{jh}\right)(1-p_{i})-\delta , & j_{0}=i, \end{array}\right.$$

we have ∇g(P)=B−Λ, where Λ is the n×n diagonal matrix where, for all i∈{1,2,…}, $\Lambda_{ii}:=\beta \sum_{h}I_{ih}f(\sum_{j}p_{j}I_{jh})\geq 0$. On the one hand, lemma 6.4 now yields $\lambda ({\left(\nabla g(P)\right)}^{\left(S\right)})\leq \lambda (B^{\left(S\right)})$; on the other hand, note that B+δI≤∇g(0)+δI, where we interpret the inequality in a componentwise sense and where we use $f^{\prime }(\sum_{j}p_{j}I_{jh})\leq f^{\prime }(0)$, since *f* is concave. Hence $B^{\left(S\right)}+\delta I\leq \nabla g(0)+\delta I$, and since $B^{\left(S\right)}+\delta I$ has only non-negative entries, appealing to the Perron–Frobenius theorem, we have $\lambda (B^{\left(S\right)})\leq \lambda (\nabla g(0))$.

Combining these inequalities and using the spectral condition in the statement of the theorem, we deduce that

$$\lambda ({\left(\nabla g(P)\right)}^{\left(S\right)})\leq \lambda (\nabla g(0))=\lambda (\beta f^{\prime }(0)W-\delta I)<0,$$

as required. ▪

When *f* is the identity—which is concave—we are effectively using the standard graph-based model, albeit with weighted edges. The inequality λ(W)β/δ<1 in theorem 6.5 then generalizes the well-known vanishing condition (2.2) found, for instance, in [8–10]. To our knowledge, previous arguments leading to this vanishing condition for graphs were not completely rigorous. Theorem 6.5 provides a new, rigorous justification for this spectral bound in the case of traditional mean field graph models.

A straightforward adaptation of the proof of theorem 6.5 yields the following global asymptotic stability result for the more general partitioned model.

**Theorem 6.6.**

*Suppose all* $f_{k}$ *are concave. If*

$$\lambda \left(\sum_{k=1}^{K}f_{k}^{\prime }(0)W^{\left(k\right)}\right)\frac{\beta }{\delta }<1,$$

*then* $0\in R^{n}$ *is a globally asymptotically stable equilibrium of* (*4.1*), *with* $g_{i}$ *defined in* (*6.2*).

## 7. Simulations to test the spectral condition

**Figure 5.**
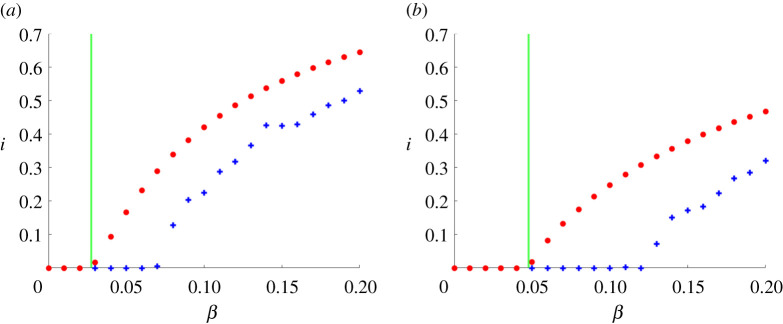
(*a*) Infection function based on 2log⁡(1+x). (*b*) Infection function based on arctan(x). For different choices of infection strength *β* (horizontal axis), we show the proportion of infected individuals at time t=150 (vertical axis) for the mean field approximation (4.1) with (6.2) in red circles and for the individual-level stochastic model (3.2) in blue crosses. The spectral bound arising from our analysis is shown as a green vertical line.(Online version in colour.)

**Figure 6.**
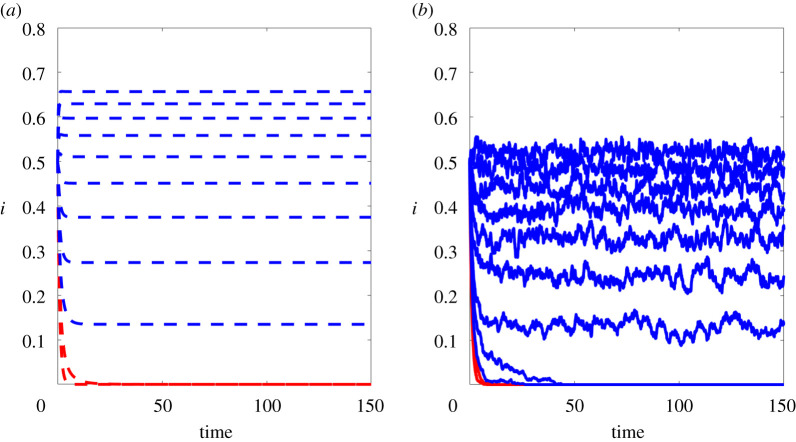
Results with the logarithmic infection function. (*a*) Proportion of infected individuals using the mean field model. (*b*) Proportion of infected individuals using the individual-level model. From bottom to top, the *β* values used were β∈{(0.02)k | k∈{0,…,10}}. Cases where *β* is below the spectral bound are coloured in red.(Online version in colour.)

**Figure 7.**
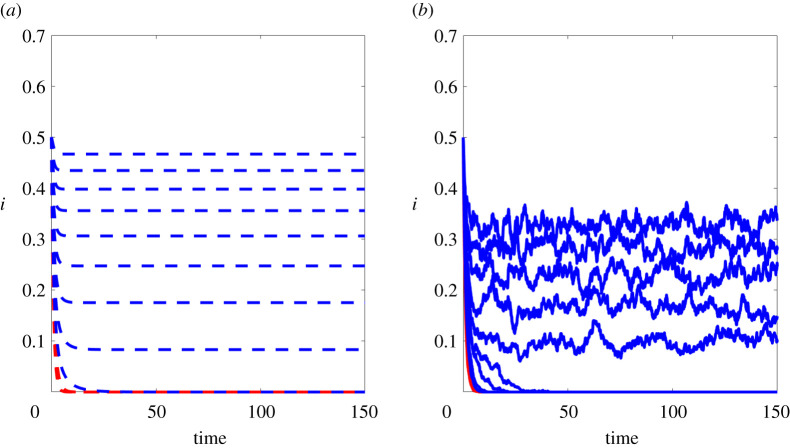
Results with the arctan infection function. (*a*) Proportion of infected individuals using the mean field model. (*b*) Proportion of infected individuals using the individual-level model. From bottom to top, the *β* values used were β∈{(0.02)k | k∈{0,…,10}}. Cases where *β* is below the spectral bound are coloured in red. (Online version in colour.)

We now show the results of experiments that test the sharpness of our spectral vanishing condition. Here, we used the concave functions f(x)=2log⁡(1+x) (on figures 5*a* and 6) and f(x)=arctan(x) (on figures 5*a* and 7) to construct partitioned models with $f_{1}(x)=x$ and $f_{k}(x)=f(x)$ for all k≥2. We fixed a hypergraph with n=400 nodes, 400 edges, 200 hyperedges of size 3, 100 hyperedges of size 4 and 50 hyperedges of size 5. At time zero, each node was infected with independent probability $i_{0}=0.5$ and we used a recovery rate of δ=1. In addition to the mean field ODE, we also simulated the microscale model, averaged over five runs, using the discretization scheme described in §5b, with Δt=0.1.

In figure 5, the circles (red) show the corresponding proportion of infected individuals according to the mean field model, $\sum_{i}p_{i}(t)/n$, at time t=150, for a range of different *β* between 0 and 0.2: β∈{(0.01)k | k∈{0,…,20}}. The crosses (blue) show the corresponding proportion of infected individuals from the microscale model, $\sum_{i}X_{i}(t)/n$, at time t=150. The vertical green line represents the critical value $\beta_{c}:=\delta /\lambda (\sum_{k=1}^{K}f_{k}^{\prime }(0)W^{\left(k\right)})$ (we have $\beta_{c}\approx 0.0265$ on the left and $\beta_{c}\approx 0.0490$ on the right).

For the mean field model, we know from theorem 6.6 that $\beta <\beta_{c}$ guarantees global stability of the zero-infection state. We see that $\beta_{c}$ also lies close to the threshold beyond which extinction of the disease is lost in the mean field model. For the individual-level stochastic model, theorem 8.3 below shows that $\beta <\beta_{c}$ is also sufficient for eventual extinction of the disease. This is consistent with the results in figure 5.

In figures 6*a* and 7*a*, we show individual trajectories of the proportion of infected individuals, $\sum_{i}p_{i}(t)/n$, according to the mean field model, for a range of *β* values. For the same range of *β* values, the plots on the right of these figures show the corresponding proportion of infected individuals from the microscale model, $\sum_{i}X_{i}(t)/n$. The curves are coloured in red if the spectral vanishing condition $\beta <\beta_{c}$ is satisfied. We see qualitative agreement between the mean field and individual-level models, and extinction for the *β* values below the spectral threshold.

Having derived and tested spectral conditions that concern extinction of the disease at the mean field approximation level, in the next section, we study the microscale model directly.

## 8. Exact model

To proceed, we recall our setting where at time zero each node has the same, independent, probability, $i_{0}$, of being infectious; so $P(X_{j}(0)=1)=i_{0}$ for all 1≤j≤n. This implies that $n\;i_{0}$ is the expected number of infectious individuals at time zero.

We are interested in the stochastic process $\sum_{i}X_{i}(t)$, which records the number of infected individuals. Our analysis generalizes arguments in [8], which considered a stochastic SIS model on a graph with *f* as the identity map. We are able to prove results in the case of concave nonlinearites, giving insights into the behaviour of the exact model and also allowing comparison with corresponding results for the mean field approximation. Furthermore, we show in §9 how these results can be extended to the case of collective contagion models.

### (a) Extinction

Our first result shows that the spectral condition arising from the mean field analysis in theorems 6.1 and 6.5 is also relevant to the probability of extinction in the individual-level model.

**Theorem 8.1.**

*Suppose f is concave in the hypergraph infection model* (*3.1*). *Then*

$$P\left(\sum_{i}X_{i}(t)>0\right)\leq n\;i_{0}\;\exp((\beta f^{\prime }(0)\lambda (W)-\delta )t).$$

*Hence, if* $\lambda (W)f^{\prime }(0)\beta /\delta <1$ *then the disease vanishes at an exponential rate*.

**Proof.**

Consider the continuous time Markov process ${\left\{{\left(Y_{i}(t)\right)}_{t\geq 0}\right\}}_{i=1}^{n}$ taking values in $N^{n}$, with transition of states defined for every 1≤i≤n and  t≥0 by

$$\left\{ \begin{array}{l} k\to k+1,\;\text{with rate}\;\beta \;f^{\prime }(0)\sum_{j}W_{ij}Y_{j}(t),\\ k\to k-1,\;\text{with rate}\;\delta \;Y_{i}(t). \end{array}\right.$$

This new process is introduced here purely for the purpose of analysis. However, it may be interpreted as a disease model where the state of each individual is represented by a non-negative integer that indicates severity of infection. Here, exposure to highly infected individuals raises the chance of an increase in infection severity.

Suppose also that $X_{i}(0)=Y_{i}(0)$ for all 1≤i≤n. Since *f* is concave,

$$\beta \sum_{h}I_{ih}f\left(\sum_{j}X_{j}(t)I_{jh}\right)\leq \beta \sum_{h}I_{ih}f^{\prime }(0)\sum_{j}X_{j}(t)I_{jh}=\beta f^{\prime }(0)\sum_{ij}W_{ij}X_{j}(t),$$

from which we see that $Y_{i}$ stochastically dominates $X_{i}$. Hence

$$P\left(\sum_{i}X_{i}(t)>0\right)\leq P\left(\sum_{i}Y_{i}(t)>0\right)\leq \sum_{i}q_{i}(t),$$

where $q_{i}(t):=E[Y_{i}(t)]$. In terms of the Chapman–Kolmogorov equation, or chemical master equation [20], we have

$$\frac{\text{d}q_{i}(t)}{\text{d}t}=\beta f^{\prime }(0)\sum_{j}W_{ij}q_{j}(t)-\delta q_{i}(t).$$

Letting $Q(t)={\left[q_{1}(t),q_{2}(t),\ldots ,q_{n}(t)\right]}^{T}$, this linear ODE system solves to give

$$Q(t)=\exp(t(\beta f^{\prime }(0)W-\delta I))Q(0).$$

The matrix $\exp(t(\beta f^{\prime }(0)W-\delta I))$ is symmetric and has spectral radius $\exp((\beta f^{\prime }(0)\lambda (W)-\delta I)t)$. Hence, in Euclidean norm,

$$||Q(t)||\leq \exp((\beta f^{\prime }(0)\lambda (W)-I)t)||Q(0)||.$$

Since $||Q(0)||=\sqrt{n}\;i_{0}$ and, by Cauchy–Schwarz,

$$\sum_{i}q_{i}(t)\leq \sqrt{n}||Q(t)||,$$

the proof is complete. ▪

We deduce, analogously to [8], the following corollary.

**Corollary 8.2.**

*Suppose f is concave in the hypergraph infection model* (*3.1*). *Let τ denote the time of extinction of the disease and suppose* $\lambda (W)f^{\prime }(0)\beta <\delta$, *then*

$$E[\tau ]\leq \frac{\log n+1}{\delta -f^{\prime }(0)\beta \lambda (W)}.$$

**Proof.**

Using theorem 8.1,

$$\begin{array}{rl} E[\tau ] & =\int_{0}^{\infty }P(\tau >t)\;\text{d}t\\ & =\int_{0}^{\infty }P(\sum_{i}X_{i}(t)>0)\;\text{d}t\\ & \leq \frac{\log n}{\delta -f^{\prime }(0)\beta \lambda (W)}+\int_{\left(\log n)/(\delta -f^{\prime }(0)\beta \lambda (W)\right)}^{\infty }n\exp((\beta f^{\prime }(0)\lambda (W)-\delta )t)\;\text{d}t\\ & \leq \frac{\log n+1}{\delta -f^{\prime }(0)\beta \lambda (W)}. \end{array}$$

 ▪

Likewise, the partitioned case yields the following result.

**Theorem 8.3.**

*Suppose every* $f_{k}$ *is concave in the partitioned hypergraph model with infection rate* (*3.2*). *Then*

$$P\left(\sum_{i}X_{i}(t)>0\right)\leq n\;i_{0}\;\exp\left(\beta \lambda (\sum_{k=1}^{K}f_{k}^{\prime }(0)W^{\left(k\right)})-\delta \right).$$

*Hence, if* $\lambda (\sum_{k=1}^{K}f_{k}^{\prime }(0)W^{\left(k\right)})\beta /\delta <1$ *then the disease vanishes at an exponential rate*.

We also have the following analogue of corollary 7.2 on the expected time to extinction for the partitioned case.

**Corollary 8.4.**

*Suppose every* $f_{k}$ *is concave in the partitioned hypergraph model with infection rate* (*3.2*). *Let τ denote the time of extinction of the disease and suppose* $\lambda (\sum_{k=1}^{K}f_{k}^{\prime }(0)W^{\left(k\right)})\beta /\delta <1$, *then*

$$E[\tau ]\leq \frac{\log n+1}{\delta -\beta \lambda (\sum_{k=1}^{K}f_{k}^{\prime }(0)W^{\left(k\right)})}.$$

### (b) Conditions that preclude extinction

So far, we have focused on deriving thresholds that imply extinction. In this subsection, following ideas from [8], we derive a condition under which the disease will persist.

Note that our analysis does not require the graph associated with *W* to be connected. The disconnected setting is relevant, for example, when interventions have been imposed in order to limit interactions. We let Δ:=D−W denote the Laplacian, and let $\lambda_{c}(\Delta )>0$ denote the smallest non-zero eigenvalue of Δ. We also let $e_{\max}$ denote the size of the largest hyperedge.

**Definition 8.5.**

Given a hypergraph H, a function $f:R_{+}\to R_{+}$ and a subset of the nodes S⊂V, let

$$E(S,f):=\sum_{i\in S}\sum_{h\in E}I_{ih}f\left(\sum_{j\in S^{c}}I_{jh}\right),$$

where $S^{c}:=V\setminus S$ is the complement of S. Also define for integer 1≤m≤⌊n/2⌋

$$\eta (H,m,f):=\inf\left\{\frac{E(S,f)}{|S|}\;|\;\;1\leq |S|\leq m\right\},$$

and let η(H,m):=η(H,m,Id).

Note that when S consists of those nodes for which $X_{i}(t)=0$, we can write the infection transition rate of $\sum_{i=1}^{n}X_{i}(t)$ as βE(S,f). More generally, βE(S,f) may be regarded as the rate at which nodes in the set S may be infected by nodes in the remainder of the network. When f=Id and m=⌊n/2⌋, η(H,m) is the Cheeger constant, or isoperimetric number, associated with the weighted graph induced by $W=II^{T}$. We may also regard η(H,m,f) as the smallest average infection rate over all subsets consisting of no more than half of the network.

The next theorem gives a probabilistic lower bound on the time to extinction.

**Theorem 8.6.**

*Recall that τ denotes the hitting time of the state* 0 *for the process* ${\left(\sum_{j}X_{j}(t)\right)}_{t\geq 0}$ *in the hypergraph model* (*3.1*). *If f is concave and* $\lambda_{c}(\Delta )>(2((e_{\max}-1)/f(e_{\max}-1)))(\delta /\beta )$, *then*

$$P\left(\tau >\frac{\left\lfloor r^{-m+1}\right\rfloor }{2m}\right)\geq \frac{1-r}{e}(1+O(r^{m})),$$

*where* $r:=(e_{\max}-1)\delta /f(e_{\max}-1)\;\beta \;\eta (H,m)<1$ *and* m:=⌊n/2⌋.

From standard Cheeger inequalities [21], we know that the Cheeger constant of the graph induced by *W* satisfies $2\eta (H,m)\geq \lambda_{c}(\Delta )$; hence, using the assumptions on $\lambda_{c}(\Delta )$ in the theorem, we see that r<1 indeed.

In order to prove this result, we introduce the following lemma.

**Lemma 8.7.**

*If f is concave and non-decreasing, then*

$$\frac{f(e_{\max}-1)}{e_{\max}-1}\eta (H,m)\leq \eta (H,m,f).$$

**Proof.**

The proof is immediate once we see that, by concavity of *f*, for all $x\in \{0,\ldots ,e_{\max}-1\}$

$$\frac{f(e_{\max}-1)}{e_{\max}-1}x\leq f(x).$$

 ▪

Now, to prove theorem 8.6 consider the Markov process ${\left(Z(t)\right)}_{t\geq 0}$ valued in {0,…,m}, with transition of states given by

$$\left\{ \begin{array}{l} k\to k+1\;\text{with transition rate}\;k\beta \frac{f(e_{\max}-1)}{e_{\max}-1}\eta (H,m),\\ k\to k-1\;\text{with transition rate}\;k\delta . \end{array}\right.$$

This Markov process is stochastically dominated by $\left({\left(\sum_{i=1}^{n}X_{i}(t)\right)}_{t\geq 0}\right)$, which has the same downward transition rate and an upward transition rate that is at least as large

$$\begin{array}{rl} k\beta \frac{f(e_{\max}-1)}{e_{\max}-1}\eta (H,m) & \leq k\beta \eta (H,m,f)\\ & \leq \beta E(S,f), \end{array}$$

where $S:=\{i\in \{1,2,\ldots ,n\}\;|\;X_{i}(t)=0\}$. Thus, to show theorem 8.6 it suffices to find a suitable lower bound for $P(\hat{\tau }>\left\lfloor r^{-m+1}\right\rfloor /2m)$, where $\hat{\tau }$ is the hitting time of 0 for the process ${\left(Z(t)\right)}_{t\geq 0}$. This follows by applying theorem 4.1 of [8] to the process ${\left(Z(t)\right)}_{t\geq 0}$.

## 9. Collective contagion models

We now consider the collective contagion models from [5–7], where infection only starts spreading within a hyperedge once a threshold number of infectious nodes in that hyperedge has been reached. As discussed in §2b, collective contagion models can be represented by nonlinear functions of the form f(x):=max{0,x−c} for some c>0, or $f(x):=c_{2}1(x\geq c_{1})$ for some $c_{1},c_{2}>0$. In these cases, it is obvious that the zero-infection state for the mean field approximation is locally asymptotically stable (and, indeed, theorem 6.1 applies). However, because the functions are not concave, the theory found in §8 for the exact model does not directly apply. Nonetheless, we can still derive similar spectral conditions for the vanishing of the disease by finding concave functions which serve as upper bounds for *f*. For instance using $c_{2}1(x\geq c_{1})\leq \frac{c_{2}}{c_{1}}x1(x\leq c_{1})+c_{2}1(x\geq c_{1})$, the bounds in theorem 8.1 and corollary 8.2 lead to the following result.

**Theorem 9.1.**

*Suppose that* $f(x):=c_{2}1(x\geq c_{1})$ *for some* $c_{1},c_{2}>0$. *Then*

$$P\left(\sum_{i}X_{i}(t)>0\right)\leq n\;i_{0}\;\exp\left(\beta \frac{c_{2}}{c_{1}}\lambda (W)-\delta \right).$$

*In particular, if* $\lambda (W)<(c_{1}/c_{2})\delta /\beta$, *then the disease asymptotically vanishes with exponential decay and the extinction time τ satisfies*

$$E[\tau ]\leq \frac{\log n+1}{\delta -\beta \lambda (W)c_{2}/c_{1}}.$$

Likewise note that $\max\{0,x-c\}\leq (e_{\max}-1-c)/(e_{\max}-1)x,$ where we recall that $e_{\max}$ is the largest size of a hyperedge of H. Hence, we deduce the following result.

**Theorem 9.2.**

*Suppose that* f(x):=max{0,x−c} *for some* c>0. *Then*

$$P\left(\sum_{i}X_{i}(t)>0\right)\leq n\;i_{0}\;\exp\left(\beta \frac{e_{\max}-1-c}{e_{\max}-1}\lambda (W)-\delta \right).$$

*In particular, if*

$$\lambda (W)<\left(\frac{e_{\max}-1}{e_{\max}-1-c}\right)\frac{\delta }{\beta },$$

*then the disease asymptotically vanishes with exponential decay and the extinction time τ satisfies*

$$E[\tau ]\leq \frac{\log n+1}{\delta -\beta ((e_{\max}-1-c)/(e_{\max}-1))\lambda (W)}.$$

## 10. Discussion

In this work, we derived spectral conditions that control the spread of disease in an SIS model on a hypergraph. The conditions have the general form

10.1 $$\frac{\beta \;\lambda (W)\;c_{f}}{\delta }<1,$$

where $c_{f}>0$ is a constant depending on the function *f* that determines the nonlinear infection rate within a hyperedge.

We note that in the special case where (i) the hypergraph is an undirected graph and hence *W* becomes the binary adjacency matrix and (ii) we have linear dependence on the number of infectious neighbours for the infection rate of a node, so *f* is the identity function, the condition (10.1) reduces to the well-known vanishing spectral condition studied in, for example, [8–10].

There are two important points to be made about the general form of (10.1). First, the hypergraph structure appears only via the presence of the symmetric matrix $W\in R^{n\times n}$. Recall that $W_{ij}$ records the number of times that *i* and *j* both appear in the same hyperedge. Such weighted but *pairwise* information is all that feeds into this spectral threshold. On a positive note, this implies that useful predictions can be made about disease spread on a hypergraph without full knowledge of the types of hyperedge present and the distribution of nodes within them. (For example, when collecting human interaction data it is more reasonable to ask an individual to list each contact and state how many different ways they interact with that contact than to ask an individual to list all hyperedges they take part in.) However, this observation also raises the possibility that more refined analysis might lead to sharper bounds, perhaps at the expense of simplicity and interpretability.

Our second point is that the new vanishing condition (10.1) neatly separates into three aspects:

(i) The biologically motivated infection parameter, *β*.
(ii) The interaction structure, captured in λ(W).
(iii) The coefficient $c_{f}$ that arises from modelling the nonlinear infection process. For instance, theorems 6.1 and 6.5 have $c_{f}=f^{\prime }(0)$. In the collective contagion model case $f(x)=c_{2}1(x\geq c_{1})$, theorem 9.1 indicates that we can take $c_{f}=c_{2}/c_{1}$.

We may view *β* as an invariant biological constant that reflects the underlying virulence of the disease and is not affected by human behaviour. The factor λ(W), which arises from the interaction structure, will be determined by regional and cultural issues, including population density, age demographics, typical household sizes and the nature of prevalent commercial and manufacturing activities. Interventions, including full or partial lockdowns, could be modelled through a change in λ(W). The third factor, $c_{f}$, is strongly dependent upon human behaviour and may be adjusted to reflect individual-based containment strategies such as social distancing, mask wearing or more frequent hand washing.

This work has focused on modelling, analysis and interpretation at the abstract level, concentrating on the fundamental question of disease extinction. Having developed this theory, it would, of course, now be of great interest to perform practical experiments using realistic interaction and infection data, with the aim of

— calibrating model parameters,
— testing hypotheses about the appropriate functional form of the infection rate,
— testing the predictive power of the modelling framework, especially in comparison with simpler homogeneous mixing and pairwise interaction versions,
— quantifying the effect of different interventions.

## Data accessibility

Code to run the experiments reported here is available at https://www.maths.ed.ac.uk/dhigham/algfiles.html.
